# Assessment of Serum Magnesium Mean Levels at Pre- and Postmagnesium Sulfate Uptake for Eclampsia Prevention: A Cross-Sectional Study

**DOI:** 10.1155/ijhy/5541651

**Published:** 2025-05-08

**Authors:** Tuan Minh Vo, Du Van Tran, Dat Quoc Nguyen, Toan Thi Nguyen, Mo Ha Nguyen, Thanh Tan Nguyen, Liem Ba Le

**Affiliations:** ^1^Department of Obstetrics and Gynecology, University of Medicine and Pharmacy at Ho Chi Minh City, Ho Chi Minh City, Vietnam; ^2^Department of Epidemiology, University of Medicine and Pharmacy at Ho Chi Minh City, Ho Chi Minh City, Vietnam; ^3^Department of Clinical Pathology, University of Medicine and Pharmacy at Ho Chi Minh City, Ho Chi Minh City, Vietnam; ^4^Department of Emergency, Hung Vuong Hospital, Ho Chi Minh City, Vietnam

**Keywords:** eclampsia, magnesium sulfate, serum magnesium level, severe preeclampsia

## Abstract

**Objective:** To assess serum magnesium mean levels in pregnant women with severe preeclampsia at three landmarks: prior to MgSO_4_ intake, 30 min, and 6 h postintake of loading dose plus maintenance dose.

**Methodology:** This cross-sectional study collected blood samples over a timeframe of 0–6 h from 64 pregnant women diagnosed with severe preeclampsia who were receiving MgSO_4_ therapy at the emergency management department of Hung Vuong Hospital, Vietnam, in the period of November 2023 to April 2024. Serum magnesium levels were measured three times in the timeframe.

**Results:** Prior to MgSO_4_ intake, the serum magnesium mean level was 0.75 ± 0.13 mmol/L. At 30 min postloading dose intake plus maintenance, the level increased to 1.65 ± 0.32 mmol/L, and at 6 h, 1.6 ± 0.34 mmol/L, where 17.2% of patients had a serum magnesium level of 2 mmol/L or higher.

**Conclusion:** There were no eclampsia incidents in patients with severe preeclampsia treated with a regimen of a loading dose of 4.5 g MgSO_4_ followed by a 1 g-hourly maintenance. Nevertheless, about 17% of participants achieved the desired threshold of 2 mmol/L, indicating a need for additional research to refine the loading and maintenance doses of MgSO_4_ for better management of severe preeclampsia in Vietnamese women.

## 1. Introduction

Preeclampsia (PE) and eclampsia are among the leading causes of maternal illnesses and fatalities worldwide. PE prevalence is estimated to account for 3%–5% of pregnancies, and its complications affect 2%–8% more of all pregnancies, causing around 63,000 maternal deaths a year globally [1]. Eclampsia continues to play a major role in maternal illness and poses huge challenges to the healthcare system and society.

The World Health Organization (WHO) identifies magnesium sulfate as a preferred agent to prevent and treat eclampsia [2], and recommends serum magnesium levels between 2 and 3.5 mmol/L for effective prevention [2–5]. Nonetheless, the optimal strategy for preventing eclampsia and minimizing side effects is not yet established as variations in body structure and weight among different populations can influence serum magnesium levels; toxicity may arise when levels exceed 3.5 mmol/L [6, 7]. In Vietnam, the MgSO_4_ dosage guidance comes primarily from international guidelines which are not fully matched with the physical nature and body weight of the Vietnamese population.

Hung Vuong Hospital, which accommodates pregnant women from southern regions, has 900 patient beds and manages around 35,000 births each year. There were 330 severe PE cases admitted to the emergency management department in 2022 [8]. Prior to 2023, Hung Vuong Hospital administered intravenous loading doses of 2–4 g of magnesium sulfate, with a preferred dose of 3 g, followed by a maintenance dose of 1-2 g per hour, with 1 g per hour preferred [9]. Clinical practice and medical literature, however, revealed that this dosing formula did not adequately prevent eclampsia in severe patients. Since September 20, 2023, the Vietnam Ministry of Health has modified the loading dose to 4–6 g of magnesium sulfate, with 4.5 g preferred, and maintains the maintenance dose at 1 g per hour [10]. Consequently, there is a need for a study to evaluate the mean serum magnesium level over time, particularly in the context of the hospital's new guidelines.

This research aimed to determine the serum magnesium mean levels in pregnant women with severe PE at Hung Vuong Hospital at three landmarks: prior to MgSO_4_ intake (baseline), 30 min, and 6 h postloading dose plus maintenance dose. The other purpose of this study was to evaluate the effectiveness of the new protocol in achieving serum magnesium levels of ≥ 2 mmol/L to prevent eclampsia in severe PE women as stated in the WHO 2011 guidelines [2].

## 2. Methodology

### 2.1. Study Design, Location, and Sample Size

A cross-sectional study was carried out at Hung Vuong Hospital. The sampling was processed during the study period, and the sample size was determined with a population mean formula, resulting in a minimum sample of 62 patients (*σ* = 0.2, as per Pascoal) [11]. Ultimately, 64 pregnant women admitted to the emergency management department between November 2023 and April 2024 were recruited. Inclusion criteria encompassed pregnant women aged more than 18 who were diagnosed with severe PE by MOH guidelines, with no previous MgSO_4_ intake [12]. PE diagnosis required at least one of the following conditions: systolic blood pressure ≥ 160 mmHg or diastolic pressure ≥ 100 mmHg, proteinuria ≥ 5 g/24 h or dipstick readings ≥ 3+ from two random urine samples, oliguria (less than 500 mL/24 h), creatinine blood levels > 1.3 mg/dL, elevated liver enzymes (AST or ALT), high uric acid, fetal growth restriction, headache or visual disturbances, and pain in the right upper quadrant or epigastric areas. Exclusion criteria comprised prior use of MgSO_4_, and such contraindications as myasthenia gravis, heart blockage, severe renal impairment (urine output ≤ 100 mL/4 h), hepatic encephalopathy, or allergy to MgSO_4_.

Each severe PE participant was given a loading dose of 4.5 g MgSO_4_ intravenously and time counted as zero, followed by a maintenance dose of 1 g-hourly. Serum magnesium levels were measured at time zero (baseline preloading intake), at 30 min, and 6 h (postloading intake plus maintenance). Blood samples were taken from the arm not for IV line, barcoded, properly stored, and assessed by laboratory technicians utilizing the Cobas 8000 automated biochemistry analyzer machine.

### 2.2. Variables and Measurement

The prime variable was serum magnesium levels at baseline, 30 min, and 6 h postloading intake plus maintenance dose. Clinical data also included glucose level, liver enzymes (AST and ALT), creatinine, proteinuria, blood urea nitrogen, and uric acid level at admission, alongside sociodemographic information gathered through structured patient interviews.

### 2.3. Statistical Analysis

Descriptive statistics including percentage, mean, and median were employed to evaluate variables within the study groups. Preliminary relationships between serum magnesium levels < 2 mmol/L and other factors were assessed with bivariate analysis, followed by multivariable logistic regression at *p* < 0.05.

## 3. Results

The mean age of participants was 33.5 years, with a standard deviation of 5.7, and ranged between 21 and 48. About two-thirds of participants had a prepregnancy BMI of 23 or higher, and the majority did not have any PE in past pregnancies. More than half of the women were diagnosed with PE at 34 weeks or more.  Table 1 presents an overview of the basic data.

Table 2 details serum magnesium levels at three landmarks: 0 (baseline), 30 min, and 6 hours (postintake).

The baseline magnesium levels were low at 0.68 mmol/L, far from the target level of 2 mmol/L. At the 30-min landmark, the mean level increased to 1.65 mmol/L and firmly around 1.6 mmol/L at the 6 h point. All magnesium means levels failed to reach the target level of 2 mmol/L.

**Figure 1:**
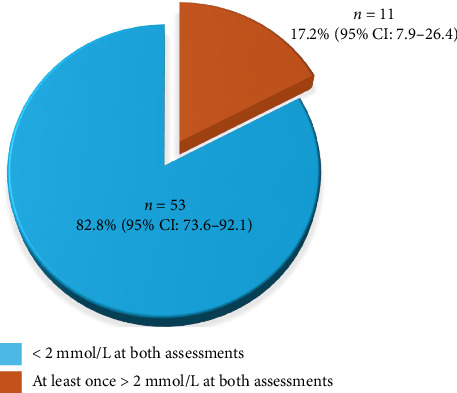
Proportion of pregnant women reaching the target magnesium level.

To evaluate eclampsia prevention effects, we rated serum magnesium levels at 30 min and 6 h into two groups: (1) Serum magnesium levels less than 2 mmol/L at both postintake landmarks and (2) serum magnesium levels 2 mmol/L or higher at one or both postintake landmarks. Among the 64 participants, about 11% of participants achieved serum magnesium levels of 2 mmol/L or higher at the 6 h point.  Figure 1 demonstrates the distribution of the two groups.

To understand the relationship between several factors and lower magnesium levels, 24 variable pairs were assessed with univariate analysis, and five pairs (*p* < 0.05) were selected for multivariate analysis to control confounders and cross-impacts.  Table 3 provides a look at the five factors in multivariate analysis.

By multivariable regression analysis on the adjusted prevalence odds ratio (POR^∗^), in the group of magnesium levels > 2 mmol/L, overweight/obese women prior to pregnancy had a higher chance of 37.7 times (95% CI: 3.01–471.98) of reaching the target level than normal/underweight women (*p*^∗^ < 0.01). In contrast, still, in that group, pregnant women with serum uric acid ≥ 360 mmol/L at hospitalization had a much lower chance of reaching the target level by one-tenth of time (95% CI: 0.01–0.96) than those with uric acid level < 360 mmol/L (*p*^∗^ < 0.05). Furthermore, pregnant women with a gestational age of 34 weeks or more at severe PE diagnosis had a higher chance to obtain the target level 10 times only (95% CI: 1.25–79.67) than those with lower gestational age (*p* < 0.05).

## 4. Discussion

No eclampsia incidents were found in severe PE patients who were treated with magnesium sulfate by the protocol of loading dose plus maintenance. Nevertheless, as indicated by our study at Hung Vuong Hospital for magnesium level effect to prevent eclampsia at 2 mmol/L, about 17.2% of participants reached that level threshold after loading dose intake.

Our research results are compared with previous studies (Table 4). At baseline point, the serum magnesium mean level was 0.75 ± 0.13 mmol/L, very close to Handwerker's findings (0.76 ± 0.05 mmol/L) [15], Chissell (0.8 ± 0.12 mmol/L) [14], and Abbade (0.74 ± 0.04 mmol/L) [16].

At the 30-min landmark, the mean level was 1.65 ± 0.32 mmol/L, higher than Abbade et al. [16] (1.48 ± 0.16 mmol/L) but lower than Chissell et al. [14] (1.9 mmol/L) and Phuapradit et al. [13] (1.97 ± 0.16 mmol/L). The discrepancy may be attributed to different protocols in which our study utilized a loading dose of 4.5 g, different from other protocols. However, the studies employing a nearly similar loading dose of 4 g produced comparable levels as 1.73 ± 0.14 mmol/L in Handwerker's [15] and 1.75 ± 0.3 mmol/L in Pascoal et al.'s [11].

At the six-hour landmark, the mean level was 1.6 ± 0.34 mmol/L, below the mean levels of Handwerker [15] (2.29 ± 0.25 mmol/L) and Pascoal et al. [11] (1.9 ± 0.6 mmol/L), likely due to their higher loading and maintenance doses. In addition, a Vietnamese study conducted 17 years ago by Hoang and Le [17] used 4 g MgSO_4_ for loading dose plus 1 g-hourly maintenance and reported a lower serum magnesium mean level of 1.22 mmol/L.

Our study indicated that serum magnesium mean level increased from 0.75 mmol/L at baseline to approximately 1.65 mmol/L at 30 min landmark and remained stable at 6 h using the updated regimen. This outcome corresponds with observations by Okusanya and colleagues in a meta-analysis on severe PE pregnant women reporting similar findings from a compatible regimen [18]. They noted that serum magnesium levels significantly doubled within 30 min (1.48–1.70 mmol/L), while mean levels remained stable during subsequent hours. Serum levels at 8, 12, and 24 h also stayed within comparable ranges, without exceeding 2.0 mmol/L. In a separate study by Phuapradit [13] using a 5 g loading dose plus continuous maintenance infusion of 1 g/hour, serum magnesium levels sharply elevated from 0.95 to 1.97 mmol/L after 30 min, followed by a gradual decrease during the first hour before stabilizing between 2.20 and 2.42 mmol/L. Therefore, in our research, we opted to measure serum magnesium sulfate levels at three key points: before intake, 30 min, and 6 h after intake, providing a cost-effective approach to monitoring serum magnesium levels up and down.

Factors leading to a serum magnesium level of less than 2 mmol/L following treatment (below the therapeutic threshold) may be prepregnancy BMI, serum uric acid levels at hospitalization, and gestational age at PE diagnosis. Literature on magnesium pharmacokinetics discloses that serum magnesium levels are largely influenced by substance amount distribution in the body [19]. In nonpregnant subjects, intravenous magnesium sulfate circulation depends on variables such as BMI and renal clearance rates. In addition, studies on animals have demonstrated that magnesium ions can cross the placenta and accumulate in fetal tissues, resulting in reduced maternal magnesium levels [20].

### 4.1. Applicability

Our research is a front-runner in assessing serum magnesium levels following the introduction of an updated MgSO_4_ protocol at Hung Vu'o'ng Hospital, which includes a 4.5 g loading dose and maintenance dose of 1 g/hour. This provides clinical evidence regarding serum magnesium levels while implementing the updated regimen. The findings indicate that the likelihood of achieving a serum level of ≥ 2 mmol/L after the loading dose is relatively low among Vietnamese women. The current study is not new in the world but quite new to the Vietnamese people who have obvious differences in physical features, skin color, and eating habits from other populations. The study serves as a foundation for future clinical research, providing a preliminary reference point for potential interventions.

### 4.2. Restrictions

Serum magnesium level assessment was restricted to only three specific points of time in pregnant patients facing severe PE, and short monitoring for 6 h alone after the loading dose. In addition, final pregnancy outcomes were not in focus as that went beyond the study objectives. A study by Kadir Guzin et al. [21] found that intravenous magnesium sulfate prolonged bleeding time in patients with severe PE. Our study, however, did not continue to observe labor until completion, and we did not document any negative effects on pregnancy outcomes for analysis. For future research, comparing different magnesium sulfate loading doses (4, 5, and 6 g) alongside any adverse effects would be beneficial, allowing for a focus on prolonged bleeding.

## 5. Conclusion

The use of a magnesium sulfate dosing regimen with a 4.5 g loading dose followed by a 1 g/hour maintenance revealed that the serum magnesium level prior to treatment was 0.75 ± 0.13 mmol/L, increased to 1.65 ± 0.32 mmol/L at 30 min postintake, and slightly decreased to 1.6 ± 0.34 mmol/L after 6 h. No incidences of eclampsia were recorded during the study period among severe PE patients. However, when considering the minimum threshold for eclampsia prevention at 2 mmol/L, 17.2% of patients reached this target level following the loading dose. This underscores the necessity for additional research to fine-tune both the loading and maintenance doses of magnesium sulfate for severe PE treatment, and special attention should be paid to women with prepregnancy BMI indicating overweight or obesity, elevated serum uric acid levels at hospitalization, and gestational age 34 weeks or more.

## Figures and Tables

**Table 1:** Participant characteristics.

Variables	Number (*n* = 64)	Percent
Age in years^∗^: 33.5 ± 5.7 (min = 21; max = 48)		
< 35	36	56.3
≥ 35	28	43.7
Prepregnancy BMI^∗^: 24.7 ± 4.1 (min = 18.1; max = 38.1)		
≥ 23	39	61.0
< 23	25	39.1
Past PE		
None	54	84.4
Mild PE	4	6.2
Severe PE	6	9.4
Glucose level at admission (mg/dL)^∗^: 95.5 ± 27.6 (min = 58.9; max = 234)		
< 140	60	93.8
≥ 140	4	6.2
AST at admission (U/L)^∗∗^: 22 (18.1–30)		
< 50	59	92.2
≥ 50	5	7.8
ALT at admission (U/L)^∗∗^: 12 (8.4–19.8)		
< 50	60	93.8
≥ 50	4	6.2
Creatinine at admission (μmol/L)^∗^: 61.2 ± 37.7 (min = 34.4; max = 332.2)		
≤ 97	61	95.3
> 97	3	4.7
Urine protein at admission (mg/dL)^∗∗^: 2.8 (0.25–31.9)		
≤ 10	44	68.7
> 10	20	31.3
Blood urea at admission (mmol/L)^∗∗^: 3.5 (2.6–4.9)		
≤ 7.5	57	89.1
> 7.5	7	10.9
Serum uric acid at admission (mmol/L)^∗^: 377.2 ± 113.2 (min = 159; max = 644)		
< 360	32	50
≥ 360	32	50
GA at PE diagnosis		
< 34 weeks	21	32.8
≥ 34 weeks	43	47.2

*Note:* 1 mM/L = 18 mg/dL = 2 mEq/L (Mg++).

^∗^: Mean ± standard deviation.

^∗∗^: Median (interquartile range).

**Table 2:** Serum magnesium levels at three measuring landmarks.

Serum magnesium levels (mmol/L)	Mean (95% CI)	Min–max	Standard deviation
Preuptake	0.75 (0.71–0.78)	0.60–1.60	0.13
At 30 min postuptake	1.65 (1.57–1.72)	0.76–2.71	0.32
At 6 h postuptake	1.60 (1.52–1.69)	0.89–2.88	0.34

*Note:* 1 mM/L = 18 mg/dL = 2 mEq/L (Mg++).

**Table 3:** Adjusted associations between maternal serum magnesium levels and factors.

Variables	Serum magnesium level		POR (95% CI)	POR^a^ (95% CI)	*p* ^a^
	< 2 mmol/L *n* = 53 (%)	> 2 mmol/L *n* = 53 (%)			
Prepregnancy BMI					
≥ 23	37 (94.8)	2 (5.2)	10.04 (2.02–53.68)	37.70 (3.01–471.98)	**0.005**
< 23	16 (64.0)	9 (36.0)	1	1	
Serum uric acid at admission					
≥ 360 mmol/L	23 (71.8)	9 (28.2)	0.17 (0.03–0.86)	0.10 (0.01–0.96)	**0.046**
< 360 mmol/L	30 (93.8)	2 (6.2)	1	1	
GA at preeclampsia diagnosis					
≥ 34 weeks	38 (88.3)	5 (11.7)	3.04 (0.80–11.48)	9.98 (1.25–79.67)	**0.03**
< 34 weeks	15 (71.4)	6 (28.6)	1	1	
Age in years					
≥ 35	21 (75.0)	7 (25.0)	0.38 (0.10–1.44)	0.14 (0.02–1.29)	0.08
< 35	32 (88.9)	4 (11.1)	1	1	
Stillbirth					
Yes	2 (50.0)	2 (50.0)	0.18 (0.02–1.42)	0.78 (0.04–14.11)	0.87
No	51 (85.0)	9 (15.0)	1	1	

*Note:* Values in bold mean statistical significance. 1 mM/L = 18 mg/dL = 2 mEq/L (Mg++).

^a^: Adjusted by the multivariate regression model.

**Table 4:** Comparison of serum magnesium levels among studies.

Author	MgSO_4_ dose		Serum magnesium levels^∗^		
	Loading (g)	Maintenance (g)	Preuptake	At 30 min postupdate	At 6 h postuptake
Phuapradit et al. [13]	5	1	0.95 ± 0.12	1.97 ± 0.16	—
Chissell et al. [14]	6	2	0.8 ± 0.12	1.9	—
Handwerker et al. [15]	4	2	0.76 ± 0.05	1.73 ± 0.14	2.29 ± 0.25
Abbade et al. [16]	4	1	0.74 ± 0.04	1.48 ± 0.16	—
Pascoal et al. [11]	6	2	1.85 ± 0.3	1.75 ± 0.3	1.9 ± 0.6
Hoang and Le [17]	4	1	1.07	—	1.22
Our research	4.5	1	0.75 ± 0.13	1.65 ± 0.32	1.64 ± 0.34

^∗^1 mM/L = 18 mg/dL = 2 mEq/L (Mg++).

## Data Availability

The original contributions presented in this study are included in the article. Further inquiries can be directed at the corresponding author.
